# Clinical Outcomes of Ambulatory Endovascular Treatment Using 4-French and 6-French Femoral Access Strategies: The Bio4amb Multicentre Trial

**DOI:** 10.1007/s00270-020-02738-5

**Published:** 2020-12-23

**Authors:** Marianne Brodmann, Koen Deloose, Eric Steinmetz, Olivier Regnard, Jens C. Ritter, Ludovic Berger, Johannes B. Dahm, Shirley Jansen, Bibombe P. Mwipatayi, Pascal Desgranges, Klaus Hausegger, Jos C. van den Berg, on behalf of the BIO4AMB investigators

**Affiliations:** 1 Department of Angiology, Medical University Graz, Auenbruggerplatz 15, 8036 Graz, Austria; 2 Department of Vascular Surgery, Sint Blasius Hospital, Dendermonde, AZ Belgium; 3 Department of Cardiovascular and Thoracic Surgery, CHU François Mitterrand, Dijon, France; 4 Department of Vascular Surgery, Clinique Saint Joseph, Trelaze, France; 5 Department of Vascular Surgery, Fiona Stanley Hospital, Perth, Australia; 6 Vascular Surgery Department, CHU de Caen, France; 7 Department of Angiology and Cardiology, Herz- und Gefässzentrum Neu-Bethlehem, Göttingen, Germany; 8 Department of Vascular and Endovascular Surgery, Sir Charles Gairdner Hospital, Perth, WA Australia; 9 Curtin Medical School, Curtin University, Bentley, Perth, WA Australia; 10 Heart and Vascular Research Institute, Harry Perkins Institute for Medical Research, Perth, WA Australia; 11 University of Western Australia, Perth, WA Australia; 12 Perth Institute of Vascular Research, Hollywood Specialist Centre, Nedlands, Australia; 13 AP-HP, Hopital Henri Mondor, Creteil, France; 14 Department of Diagnostic and Interventional Radiology, LKH Klagenfurt, Austria; 15 Centro Vascolare Ticin, Ospedale Regionale di Lugano, Lugano, Switzerland; 16 Universitätsinstitut für Diagnostische, Interventionelle und Pädiatrische Radiologie, Universitätsspital Bern, Bern, Switzerland

**Keywords:** Peripheral vascular intervention, Ambulatory treatment, 4F, Access-site complication

## Abstract

**Purpose:**

Ambulatory peripheral vascular interventions have been steadily increasing. In ambulatory procedures, 4F devices might be particularly useful having the potential to reduce access-site complications; however, further evidence on their safety and efficacy is needed.

**Materials and Methods:**

BIO4AMB is a prospective, non-randomized mulitcentre, non-inferiority trial conducted in 35 centres in Europe and Australia comparing the use of 4F- and 6F-compatible devices. The main exclusion criteria included an American Society of Anaesthesiologists class ≥ 4, coagulation disorders, or social isolation. The primary endpoint was access-site complications within 30 days.

**Results:**

The 4F group enrolled 390 patients and the 6F group 404 patients. Baseline characteristics were similar between the groups. Vascular closure devices were used in 7.7% (4F group) and 87.6% (6F group) of patients. Patients with vascular closure device use in the 4F group were subsequently excluded from the primary analysis, resulting in 361 patients in the 4F group. Time to haemostasis was longer for the 4F group, but the total procedure time was shorter (13.2 ± 18.8 vs. 6.4 ± 8.9 min, p < 0.0001, and 39.1 ± 25.2 vs. 46.4 ± 27.6 min, *p* < 0.0001). Discharge on the day of the procedure was possible in 95.0% (4F group) and 94.6% (6F group) of patients. Access-site complications were similar between the groups (2.8% and 3.2%) and included predominantly groin haematomas and pseudoaneurysms. Major adverse events through 30 days occurred in 1.7% and 2.0%, respectively.

**Conclusions:**

Ambulatory peripheral vascular interventions are feasible and safe. The use of 4F devices resulted in similar outcomes compared to that of 6F devices.

**Supplementary Information:**

The online version of this article (10.1007/s00270-020-02738-5) contains supplementary material, which is available to authorized users.

## Introduction

The numbers of peripheral vascular interventions (PVIs) have increased substantially worldwide [1–4]. Traditionally performed on an inpatient basis, in order to free up limited resources and save costs, clinicians have to evaluate the concept of ambulatory procedures [5–7]. Although several reports have demonstrated ambulatory procedures to be safe and efficient, further evidence is needed to ensure the clinical and economic value of outpatients’ management of PVIs [7]. Furthermore, most published data available refer to 6 French (F) devices [6, 8]; however, 4F devices might be particularly useful in this setting as this low-profile approach can avoid the use of vascular closure devices (VCDs), and thus has the potential for reducing access-site-complications (ASCs) [9, 10]. The 4-EVER trial confirmed the successful use of 4F devices without the need for closure devices with only 3.3% ASCs [11].

To further assess how the use of smaller sheath sizes may affect the ambulatory treatment and the rate of ASCs, the “BIOTRONIK 4French for AMBulatory Peripheral Intervention (BIO4AMB)” study was initiated in Europe and Australia for evaluating the non-inferiority of ambulatory treatment when using 4F- versus 6F-compatible devices.

## Materials and Methods

### Study Design

BIO4AMB is a prospective, non-randomized multicentre, non-inferiority trial conducted in 35 centres in Austria, Belgium, France, Switzerland, Denmark, Germany and Australia. Equal numbers of patients in the 4F and 6F groups were intended to be enrolled at each site.

The full list of inclusion- and exclusion criteria is available at ClinicalTrials.gov:NCT03044002. Briefly, patients with infrainguinal arterial lesions suitable to be treated with an endovascular intervention in an ambulatory setting, those who were able to walk and those who signed informed consent could be included. Main exclusion criteria were American Society of Anaesthesiologists class ≥ 4, coagulation disorders, or social isolation during the first night. Patients’ assessments were performed at baseline and during the procedure, discharge, and at 30 days. The 30-day visit was scheduled as office visit, but telephone follow-ups were accepted if this was not possible.

The study was conducted according to the current version of the Declaration of Helsinki, ISO14155:2011, according to national and local requirements, and was approved by the sites’ ethic committees. Monitoring of endpoint-related data was performed for at least 25% of patients randomly selected. A clinical events committee adjudicated all endpoint-related adverse events.

### Devices and Procedures

The introducer sheaths could be selected at the physician’s discretion. In the 4F group, the Passeo-18 uncoated balloon, Passeo-18 Lux drug-coated balloon and Pulsar-18 stent could be used (all Biotronik AG, Buelach, Switzerland), but no VCD was permitted. In the 6F group, the choice of devices ought to reflect the standard of care, and the use of VCDs was optional (Supplementary Table 1). Whether a 4F- or 6F-access was used was left at the physician’s discretion, provided that the instructions for use were respected.

The access ought to be ultrasound guided, if available.

### Endpoints and Definitions

The primary endpoint was ASCs within 30 days (including haemostasis strategy failure), defined as groin hematoma (> 5 cm in diameter, visible by sonography, and haemoglobin decrease < 3 g/dL), pseudoaneurysm, groin or retroperitoneal bleeding (defined as requiring acute intervention for haemostasis, need for blood transfusions, or haemoglobin decrease > 3 g/dL), arteriovenous fistula (as evidenced by colour coded sonography), arterial dissections at the access site (visible with angiography or sonography as a membrane causing stenosis in the vessel lumen), thrombosis, or closure device-related ASC.

Secondary endpoints were procedural success (defined as no change to a larger sheath size and/or second puncture site), ambulatory failure (defined as unplanned overnight hospitalization), time to haemostasis, time to discharge, VCD failure and related complication, reinterventions, Rutherford class, and ankle brachial index (ABI) [12] at 30 days. Major adverse events (MAEs) were defined as a composite of freedom from 30-day device- or procedure-related mortality, major target limb amputation and clinically driven TLR.

### Statistical Analyses

The sample size of BIO4AMB was based on non-inferiority on the primary endpoint ASC with a non-inferiority margin of 2% assuming an ASC-rate of 5% in the 6F group and 3% in the 4F group [11, 13, 14]. A total of 792 patients (377 + 19 per group) were scheduled to be enrolled assuming a one-sided alpha of 0.025, a power of 80% and 5% dropouts.

The primary analysis consists of the modified intention-to-treat-population that excludes patients treated with a VCD in the 4F group, as no VCD is 4F compatible and using a 6F-closure device contradicts the 4F approach. The secondary analysis is based on the intention-to-treat population (Supplementary Tables 2–5). ABI-values > 1.3 were censored.

For quantitative variables, the mean values, standard deviation, and 95% confidence interval for the mean were calculated as applicable, and for qualitative variables, absolute and relative frequencies. Calculations were based on the available data. The 4F and 6F groups were compared using the Chi-square, Wilcoxon, and T tests. A *p*-value of < 0.05 was considered statistically significant.

In a posthoc-analysis, propensity score matching was performed for the factors gender, body-mass index, smoking, hypertension, diabetes, critical limb ischaemia, below-the-knee (BTK) lesions, calcification at the puncture site, antegrade versus retrograde access, previous puncture at the same site, procedure time, antiplatelet and anticoagulation agents, and for one study site that enrolled 35% of the patients and hence exceeded the common threshold of a maximum of 20–30% of patients enrolled per site (Supplementary Table 6).

In another posthoc analysis, subgroup analyses for freedom from ASC, MAE, and patients discharged on the same day of the procedure were performed for age > 65 years, females, diabetics, femoral lesions, popliteal lesions, BTK lesions, and antegrade or retrograde access. All analyses were performed using SAS version 9.4 (SAS Institute Inc, Cary, NC, USA).

## Results

Overall, 794 patients were included, wherein 390 were treated with a 4F device and 404 with a 6F device. Patients who received a VCD in the 4F group (n = 29, 7.4%) were excluded from the primary analysis (that thus encompasses 361 patients with 516 lesions), but were included in the secondary analysis (Supplementary Tables 2–5).

The baseline characteristics were similar between the groups; patients were 70 ± 11 (4F group) and 69 ± 11 years old (6F group), and 29.4% and 33.2% had diabetes. There was only a small difference between the groups regarding to hyperlipidaemia (59.3% vs. 70.5%, *p* = 0.001) and renal disease (22.4% vs. 16.1%, *p* = 0.019) (Table 1). Lesions were located in the superficial femoral artery in 57% in both the groups; BTK lesions were present more frequently in the 4F group (19.0% vs. 13.2%, *p* = 0.008). Furthermore, lesions treated with a 4F device were less calcified (moderate/heavy calcification in 40.1% vs. 47.1%, *p* = 0.002) (Table 2).

**Table 1:** Baseline patient characteristics

	4F N = 361	6F N = 404	*p*-value
Age, years	70 ± 11	69 ± 11	0.235
Male	260 (72.0)	310 (76.7)	0.136
Smoking	274 (75.9)	310 (76.7)	0.787
BMI	N = 349 6.8 ± 4.4	N = 397 7.0 ± 4.5	0.524
Hypertension	289 (80.1)	326 (80.7)	0.825
Hyperlipidaemia	214 (59.3)	285 (70.5)	**0.001**
Diabetes mellitus	106 (29.4%)	134 (33.2%)	0.258
Insulin dependent	45 (12.5)	42 (10.4)	
Renal insufficiency*	81 (22.4)	65 (16.1)	**0.026**
History of PAD	206 (57.1%)	243 (60.1%)	0.387
Previous PVI/ surgeries	167 (46.3%)	196 (48.5%)	0.533

BMI-body mass index, PAD, peripheral artery disease, PVI, peripheral vascular intervention

Data are displayed as mean ± standard deviation or n (%)

*According to site-assessment

**Table 2:** Baseline lesion characteristics

	4F *N* = 517	6F *N* = 613	*p*-value
Lesion location	*N* = 517	*N* = 613	
Common femoral	23 (4.4)	32 (5.2)	0.548
SFA	294 (56.9)	347 (56.6)	0.930
Popliteal artery	73 (14.1)	108 (17.6)	0.110
BTK	98 (19.0)	81 (13.2)	**0.008**
Other*	29 (5.6)	45 (7.3)	0.241
Calcification†	*N* = 511	*N* = 607	**0.002**
Moderate	101 (19.8)	179 (29.5)	
Heavy	104 (20.4)	107 (17.6)	
TASC classification	*N* = 512	*N* = 607	0.328
A	130 (25.4)	154 (25.4)	
B	173 (33.8)	234 (38.6)	
C	126 (24.6)	129 (21.3)	
D	83 (16.2)	90 (14.8)	
Thrombus present	*N* = 516	*N* = 609	0.380
	70 (13.6)	*N* = 72 (11.8)	

Data are displayed as mean ± standard deviation or n (%)

*4F: 9 Arteria femoralis profunda, 8 bypass grafts, 7 iliac arteries, 2 lesions extending in two vessels, 6F: 8 Arteria femoralis profunda, 10 bypass grafts, 24 iliac arteries, and one stented artery

†according to site-assessment. BTK, below-the-knee, SFA, superficial femoral artery

Vessel puncture was more frequently ultrasound guided in the 4F group (83.9% vs. 77.8%, *p* = 0.032), VCD devices were used in 87.6% (353/403) of cases in the 6F group, and additional manual compression or manual compression devices were required in 42.8% (151/353). Overall, time to haemostasis was longer in the 4F group (13.2 ± 18.8 min vs. 6.2 ± 8.9 min, *p* < 0.0001), and procedure time shorter (39.1 ± 25.2 min vs. 46.4 ± 27.6 min, *p* < 0.0001) (Table 3). Discharge on the day of procedure was nearly identical between the groups (95.0% vs. 94.6% *p* = 0.782); time to discharge was 7.9 ± 12.1 h vs. 7.8 ± 10.8 h, *p* = 0.257 (5.9 ± 2.0 h vs. 6.2 ± 2.1 h, *p* = 0.267, excluding patients with ambulatory failure). Adverse events in patients with ambulatory failures are provided in Supplementary Table 7.

**Table 3:** Procedural characteristics

	4F *N* = 361	6F *N* = 404	*p*-value
Femoral access	*N* = 366 366 (100.0)	*N* = 410 407 (99.3)	0.251
> 1 vascular access	5 (1.4)	5 (1.2)	> 0.999
Access	*N* = 366	*N* = 410	**0.002**
Antegrade	259 (70.8)	246 (60.0)	
Retrograde	107 (29.2)	164 (40.0)	
Puncture ultrasound guided	307 (83.9%)	319 (77.8%)	**0.032**
Calcification at puncture site	*N* = 366	*N* = 410	**0.001**
None	175 (47.8%)	136 (33.2%)	
Mild	136 (37.2%)	175 (42.7%)	
Moderate	39 (10.7%)	77 (18.8%)	
Heavy	16 (4.4%)	22 (5.4%)	
Vessel diameter at puncture site, mm	*N* = 366 6.47 ± 1.05	*N* = 408 6.88 ± 1.06	** < 0.001**
Previous puncture at the same site	*N* = 366 41 (11.2%)	*N* = 410 36 (8.8)	0.280
Devices used	*N* = 958	*N* = 1171	–
Plain balloon	500 (52.2)	534 (45.6)	
Drug-coated balloon	102 (10.6)	217 (18.5)	
Stent	346 (36.1)	385 (32.9)	
Rotational thrombectomy	0 (0.0)	1 (0.1)	
Atherectomy	0 (0.0)	5 (0.4)	
Scoring balloon	0 (0.0)	8 (0.7)	
Cutting balloon	2 (0.2)	8 (0.7)	
Other	8 (0.8)	13 (1.1)	
Haemostasis	*N *= 361	*N* = 403	** < 0.0001**
VCD only	0 (0)	202 (50.0)	
Compression device only	0 (0.0)	0 (0.0)	
Manual compression only	147 (40.7)	24 (5.9)	
VCD + compression device	0 (0.0)	23 (5.7)	
VCD + manual compression	0 (0.0)	91 (22.5)	
VCD + compr. device + manual compression	0 (0.0)	36 (8.9)	
Compression device + manual compression	214 (59.3)	25 (6.7)	
Other combinations	0 (0.0)	1 (0.2)	
None	0 (0.0)	1 (0.2)	
Haemostasis in ASC patients	*N* = 10	*N* = 13	**< 0.0001**
VCD only	0 (0.0)	1 (7.7)	
Manual compression only	6 (60.0)	0 (0.0)	
VCD + manual compression	0 (0.0)	3 (23.1)	
VCD + compression device + manual	0 (0.0)	8(61.5)	
compression			
Compression device + manual compression	4 (40.0)	1 (7.7)	
Manual compression time, min	*N =* 361	*N* = 402	**< 0.0001**
	9.1 ± 8.8	4.2 ± 7.4	
	[8.2;10.0]	[3.5;4.9]	0.088
Without zeros†	*N* = 361	*N* = 179	
	9.1 ± 8.8	9.6 ± 8.6	
	[8.2, 10.0]	[8.3, 10.8]	
Time to haemostasis, min	*N* = 361	*N* = 403	**< 0.0001**
	13.2 ± 18.8	6.2 ± 8.9	
	[11.3;15.2]	[5.3;7.1]	
Procedure time, min	*N* = 359	*N* = 402	**< 0.0001**
	39.1 ± 25.2	46.4 ± 27.6	
	[36.5;41.8]	[43.8;4923]	
Procedure success	358 (99.2)	400 (99.0)	> 0.999

Data are displayed as mean ± standard deviation [95%confidence interval] or n (%)

*excludes patients with VCD who did not require manual compression,

†excludes patients in whom no manual compression was performed. ASC, access-site complication, VCD, vascular closure device

At 30 days, five patients were lost-to-follow-up in the 4F group, and four patients had died in the 6F group (one sudden death, one worsening of peripheral artery disease, one cardiac arrest and one myocardial infarction). There was no significant difference in ASCs in the intention-to-treat or in the propensity-score matched cohort. ASCs occurred in 2.8% of patients in the 4F group and 3.2% of patients in the 6F group, *p* = 0.729 and p_non-inferiority_ = 0.0253 (3.3% and 2.6% in the propensity matched cohort respectively, *p* = 0.627), and were predominantly caused by groin hematoma and pseudoaneurysms (Table 4). In the 6F group, all but one ACSs occurred in patients treated with a VCDs (3.4%, 12/353).

**Table 4:** Clinical outcomes of patients for up to 30 days

	4F *N* = 361	6F *N* = 404	*p*-value
Vascular closure device complication	0 (0.0)	10 (2.5)	0.002
	*N* = 356	*N* = 401	
Access-site complications*	10 (2.8)	13 (3.2)	0.729
Groin hematoma	4 (23.5)	4 (15.4)	0.788
Pseudoaneurysm	5 (29.4)	6 (23.1)	
Groin-bleeding	1 (5.9)	2 (7.7)	
AV-fistula	0 (0.0)	0 (0.0)	
Arterial dissection	0 (0.0)	1 (3.8)	
Thrombosis	1 (5.9)	0 (0.0)	
VCD-related	0 (0.0)	0 (0.0)	
Other	6 (35.3)	13 (50.0)	
Access-site complications, matched cohort	*N* = 306 10 (3.3)	*N* = 307 8 (2.6)	0.627
	*N* = 356	*N* = 402	
Major adverse events	6 (1.7)	8 (2.0)	0.794
Procedure- or device related death	0 (0.0)	2 (0.5)†	0.501
Major target limb amputation	1 (0.3)	0 (0.0)	0.471
Clinically driven TLR	6 (1.7)	6 (1.5)	> 0.999

Data are displayed as n (%)

*could consist of several of the events below

†worsening of peripheral artery disease on day 8 and sudden death on day 16 post-procedure.

AV, arteriovenous; TLR, target lesion revascularization; VCD, vascular closure device

**Fig. 1:**
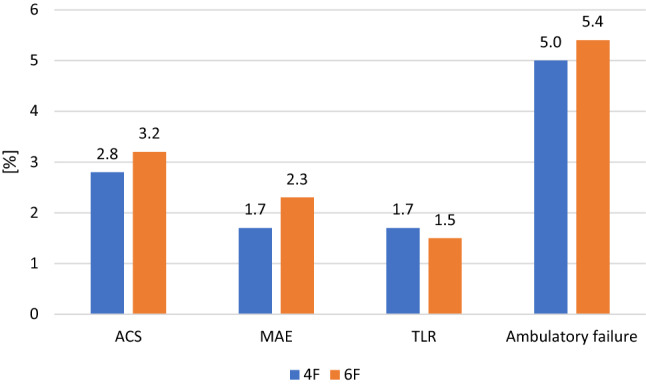
Access-site complications, major adverse events, re-interventions, and ambulatory failure with 4-French and 6-French sheaths. There was no significant difference between the groups. ASC, access-site complication, MAE, major adverse event, TLR, clinically driven target lesion revascularization

MAEs and reinterventions rates (clinically-driven target lesion revascularization) were also similar (1.7% vs. 2.0%, *p* = 0.794 and 1.7% vs.1.5%, *p* > 0.999, respectively) (Fig. 1). Furthermore, there was no significant difference in ASC, MAE and ambulatory failure in the subgroups, as specified in Table 5.

**Table 5:** Safety and efficacy of selected subgroups

	Endpoint	4F	6F	*p*-value
Age > 65 years	Freedom from ASC (subject based, %)	235 (96.7%)	247 (96.5%)	0.891
	MAE (subject based, %)	4 (1.6%)	4 (1.5%)	> 0.999
	Same-day discharge	231 (94.3%)	245 (94.6%)	0.880
Female	Freedom from ASC (subject based, %)	95 (94.1%)	89 (94.7%)	0.851
	MAE (subject based, %)	3 (3.0%)	2 (2.2%)	> 0.999
	Same-day discharge	93 (92.1%)	90 (95.7%)	0.287
Diabetics	Freedom from ASC (subject based, %)	101 (98.1%)	130 (97.0%)	0.612
	MAE (subject based, %)	1 (1.0%)	2 (1.5%)	> 0.999
	Same-day discharge	100 (94.3%)	128 (95.5%)	0.676
CFA and SFA	Freedom from ASC (subject based, %)	206 (96.7%)	223 (95.7%)	0.580
	MAE (subject based, %)	3 (1.4%)	3 (1.3%)	> 0.999
	Same-day discharge	206 (95.4%)	221 (94.8%)	0.799
Popliteal	Freedom from ASC (subject based, %)	28 (93.3%)	31 (93.9%)	0.922
	MAE (subject based, %)	0 (0%)	0 (0%)	NA
	Same-day discharge	30 (90.0%)	30 (90.9%)	0.902
BTK	Freedom from ASC (subject based, %)	36 (100%)	16 (100%)	NA
	MAE (subject based, %)	1 (2.8%)	0 (0%)	> 0.999
	Same-day discharge	34 (91.9%)	16 (100%)	0.241
Antegrade Access	Freedom from ASC (subject based, %)	249 (98.0%)	235 (97.1%)	0.503
	MAE (subject based, %)	4(1.6%)	4 (1.6%)	> 0.999
	Same-day discharge	242 (94.5%)	230 (93.9%)	0.754
Retrograde Access	Freedom from ASC (subject based, %)	97 (95.1%)	153 (96.2%)	0.658
	MAE (subject based, %)	2 (2.0%)	4 (2.5%)	> 0.999
	Same-day discharge	101 (96.2%)	152 (95.6%)	0.813

BTK, below-the-knee; CFA, common femoral artery; NA, not applicable; SFA, superficial femoral artery

**Fig. 2:**
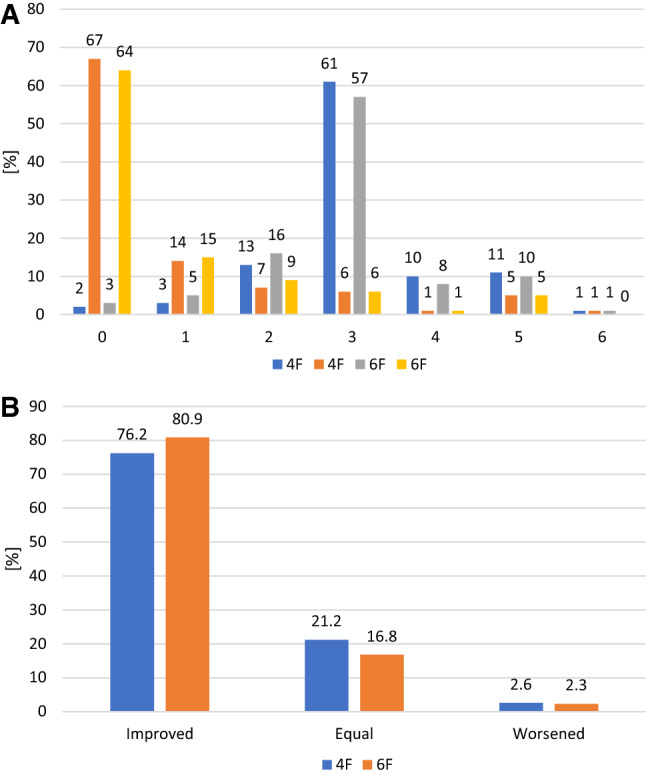
Rutherford class assessments **A** Rutherford class at baseline and 30-day follow-up** B** Change in Rutherford class at 30-day follow-up. Clinical improvement is defined as improvement of at least one Rutherford class. a data were available for 358 patients at baseline and 347 at follow-up (4F group) and 398 and 391 patients, respectively (6F group), B data were available for 345 patients (4F group) and 388 patients (6F group)

From baseline to 30 days, ABI improved from 0.75 ± 0.19 (n = 225) to 0.96 ± 0.22 (*n* = 215) in the 4F group and from 0.72 ± 0.22 (*n* = 331) to 0.96 ± 0.18 (*n* = 311) in the 6F group. Using paired data, ABI improved by 0.22 ± 0.21 (*n* = 185) and 0.25 ± 0.24 (*n* = 282), respectively. Furthermore, Rutherford class improvement was nearly identical in the groups (Fig. 2). Anticoagulation/ antiplatelet therapy from pre-procedure through 30 days is provided in Supplementary Table 8.

## Discussion

The BIO4AMB trial confirmed that the ambulatory treatment of peripheral artery disease is safe and effective regardless of whether 4F or 6F devices are used. ASC and MAE rates were low and approximately 95% of patients could be discharged on the day of the procedure (5% ambulatory failure).

### Procedure

Despite the use of VCDs in 87.4% of cases, additional treatment was required in 42.8% in the 6F group. Overall, manual compression time and time to haemostasis were longer for the 4F group, but total procedure time shorter. This is not unexpected since previous studies have reported a longer time to haemostasis with compression than with VCDs [15, 16]. Nevertheless, the manual compression time was shorter than the recommended 15 to 20 min reported previously for larger sheath sizes [15]. Furthermore, one may speculate that there is even more scrutiny in haemostasis for avoiding late complications in the ambulatory setting.

### Ambulatory Failure

Patients prefer to be discharged on the same day as the procedure [7, 17]. The use of manual compression may impact the chance to be discharged on the same day and VCDs may reduce hospital time [15, 16]. In a study by Akopian et al. [6], only 80% of patients treated with manual compression were discharged the same day versus 93% of patients treated with VCDs. However, these studies were mostly performed with sheaths larger than 4F. In our study, the rate of ambulatory failure was 5% in both the groups, and thus was within the 0–27.3% range as reported in the literature [6–8, 18].

### Access Site Complications

The use of VCDs may be associated with complications such as infection, bleeding, pseudoaneurysm, arterial laceration, arteriovenous fistulae, embolization, limb ischaemia, thrombosis, pain, dissection and nerve injury [8, 15], and no data have shown the advantages of VCDs in terms of complications such as bleeding [16].

Therefore, the primary study hypothesis for providing the non-inferiority of the 4F group compared to the 6F group, considered a higher ASC-rate in the 6F group using VCDs. However, the outcomes were nearly identical in the groups (2.8% vs. 3.2%), as the 6F group had better outcomes than the predicted 5% ASC-rate. This is likely due to improvements in VCDs, the recommended ultrasound guidance, and the fact that participating centres were experienced high-volume centres [19, 20]. Thus, the study hypothesis failed slightly (*p*_non-inferiority_ = 0.0253 instead of < 0.025) as the sample size was too low being based on a 2% margin of an expected ASC rate of 5% in the 6F group, which turned out to be lower than expected.

The outcomes might have also been biased by the fact that VCD use was not permitted in the 4F group. Meanwhile, the French guidelines [21] recommend for devices ≤ 7F to decide on the use of VCD based on factors that may be associated with impaired haemostasis such as obesity, coagulation disorder, and physician’s experience.

While the comparison of outcomes to other studies is hampered through different patient population, different scrutiny in monitoring, and different definitions of ASCs, it can still be concluded that the ASC-rate of 2.8% in the 4F group and 3.2% in the 6F group (3.3% and 2.6% in the matched cohort, respectively) is low and consistent with the 3.3% rate observed in the 4-Ever trial [11], the 3.5% rate in the Vascular Quality Initiative [14], the 0–3% major haematomas reported in a systematic review [7], and lower than the 11.5% rate reported in a retrospective single centre study [22], or the 12% of hematoma and closure device failure reported by Albert et al. [8].

### Risk Groups

With our subgroup analyses in elderly, females, diabetics, femoral, popliteal, and below-the-knee lesions, as well as for antegrade access, we have demonstrated that even in these high-risk groups ambulatory treatment is feasible with a low risk of complications, allowing an early return to home for a broad patient population.

## Limitations

This study has several limitations. It is not randomized; however, the primary endpoint was tested for potential confounding effects using propensity matching and did not reveal a significant difference between the groups (ASC-rate of 3.3% vs. 2.6% in the matched cohort). The comparison to other studies is limited owing to different definitions of ASCs and the fact that our data were monitored, whereas other data such as from the vascular surgery’s Vascular Quality Initiative database are self-reported [14]. Furthermore, no VCDs were permitted in the 4F group which does not reflect routine use and might have resulted in biased outcomes. Positive is the high follow-up compliance rate. We did not report on health economic aspects which are relevant, particularly considering the financial impact of the use of VCDs, procedure time, discharge time, and quality of life, as these data will be the subject of an upcoming publication.

## Conclusions

In summary, ambulatory treatment is a valid and safe option for endovascular treatment of lower-extremity peripheral arterial disease. 4F-compatible devices show similar short-term safety when compared to the well-established 6F devices and are a valid alternative based on patients’ needs and physicians’ preferences, while avoiding the additional need for VCDs. Further studies, including health economic aspects, are needed for better defining the appropriate patient population that benefits most from the ambulatory procedures and a minimized hospital stay, and for determining the cost-effectiveness of 4F-compatible devices compared to 6F-compatible devices.

## Electronic supplementary material

Below is the link to the electronic supplementary material.

Supplementary material 1 (DOCX 39 kb)
