# Correction to: Clinical Outcomes of Ambulatory Endovascular Treatment Using 4-French and 6-French Femoral Access Strategies: The Bio4amb Multicentre Trial

**DOI:** 10.1007/s00270-021-02827-z

**Published:** 2021-04-08

**Authors:** Marianne Brodmann, Koen Deloose, Eric Steinmetz, Olivier Regnard, Jens C. Ritter, Ludovic Berger, Johannes B. Dahm, Shirley Jansen, Bibombe P. Mwipatayi, Pascal Desgranges, Klaus Hausegger, Jos C. van den Berg, on behalf of the BIO4AMB investigators, Olivier Regnard, Marianne Brodmann, Koen Deloose, Jens Carsten Ritter, Ludovic Berger, Johannes Dahm, Shirley Jansen, Bibombe Patrice Mwipatayi, Joseph Touma, Eric Ducasse, Antoine Millon, Sébastien Veron, Raphael Coscas, Eric Steinmetz, Fabrice Schneider, Lieven Maene, Bahaa Nasr, Gilles Miltgen, Vikram Puttaswamy, Jürgen Torsten Verbist, Jonathan Sobocinski, Armand Bourriez, Mark Jackson, Laurent Casbas, Didier Paneau, Isabelle Bayens, Klaus Hausegger, David Lambrechts, Adrien Kaladji, Flemming Randsbaek, Pierre Jules Delannoy, Manfred Spanger, Jos C. van den Berg

**Affiliations:** 1 Department of Angiology, Medical University Graz, Auenbruggerplatz 15, 8036 Graz, Austria; 2 Department of Vascular Surgery, Sint Blasius Hospital, Dendermonde, AZ Belgium; 3 Department of Cardiovascular and Thoracic Surgery, CHU François Mitterrand, Dijon, France; 4 Department of Vascular Surgery, Clinique Saint Joseph, Trelaze, France; 5 Department of Vascular Surgery, Fiona Stanley Hospital, Perth, Australia; 6 Vascular Surgery Department, CHU de Caen, France; 7 Department of Angiology and Cardiology, Herz- und Gefässzentrum Neu-Bethlehem, Göttingen, Germany; 8 Department of Vascular and Endovascular Surgery, Sir Charles Gairdner Hospital, Perth, WA Australia; 9 Curtin Medical School, Curtin University, Bentley, Perth, WA Australia; 10 Heart and Vascular Research Institute, Harry Perkins Institute for Medical Research, Perth, WA Australia; 11 University of Western Australia, Perth, WA Australia; 12 Perth Institute of Vascular Research, Hollywood Specialist Centre, Nedlands, Australia; 13 AP-HP, Hopital Henri Mondor, Creteil, France; 14 Department of Diagnostic and Interventional Radiology, LKH, Klagenfurt, Austria; 15 Centro Vascolare Ticin, Ospedale Regionale Di Lugano, Lugano, Switzerland; 16 Universitätsinstitut Für Diagnostische, Interventionelle Und Pädiatrische Radiologie, Universitätsspital Bern, Bern, Switzerland

## Cardiovasc Intervent Radiol (2020). https://doi.org/10.1007/s00270-020-02738-5

The original version of this paper did not contain a list of Bio4amb investigators. The purpose of this correction is to acknowledge the contribution of all investigators who participated in the study. The ESM (Electronic Supplementary Material) file of the original article has been corrected.

**Contributors: Bio4amb investigators:** Olivier Regnard, Marianne Brodmann, Koen Deloose, Jens Carsten Ritter, Ludovic Berger, Johannes Dahm, Shirley Jansen, Bibombe Patrice Mwipatayi, Joseph Touma, Eric Ducasse, Antoine Millon, Sébastien Veron, Raphael Coscas, Eric Steinmetz, Fabrice Schneider, Lieven Maene, Bahaa Nasr, Gilles Miltgen, Vikram Puttaswamy, Jürgen Torsten Verbist, Jonathan Sobocinski, Armand Bourriez, Mark Jackson, Laurent Casbas, Didier Paneau, Isabelle Bayens, Klaus Hausegger, David Lambrechts, Adrien Kaladji, Flemming Randsbaek, Pierre Jules Delannoy, Manfred Spanger, Jos C. van den Berg.

