# Supplementary material for: Clinical Outcomes of Ambulatory Endovascular Treatment Using 4-French and 6-French Femoral Access Strategies: The Bio4amb Multicentre Trial
Source: Cardiovasc Intervent Radiol. 2020 Dec 23;44(5):689–97. doi: 10.1007/s00270-020-02738-5 (PMC8060188; doi:10.1007/s00270-020-02738-5)
Supplement: Supplementary file 1 — Supplementary material 1 (DOCX 39 kb) [file 270_2020_2738_MOESM1_ESM.docx]

**Supplementary material**

**Clinical Outcomes of Ambulatory Endovascular Treatment Using 4-French and 6-French Femoral Access Strategies: The Bio4amb Multicentre Trial**

**Contributors: Bio4amb investigators:** Olivier Regnard, Marianne Brodmann, Koen Deloose, Jens Carsten Ritter, Ludovic Berger, Johannes Dahm, Shirley Jansen, Bibombe Patrice Mwipatayi, Joseph Touma, Eric Ducasse, Antoine Millon, Sébastien Veron, Raphael Coscas, Eric Steinmetz, Fabrice Schneider, Lieven Maene, Bahaa Nasr, Gilles Miltgen, Vikram Puttaswamy, Jürgen Torsten Verbist, Jonathan Sobocinski, Armand Bourriez, Mark Jackson, Laurent Casbas, Didier Paneau, Isabelle Bayens, Klaus Hausegger, David Lambrechts, Adrien Kaladji, Flemming Randsbaek, Pierre Jules Delannoy, Manfred Spanger, Jos C. van den Berg

**Supplementary Table 1: Permitted use of devices in the 4F and 6F groups**

| **Device** | **4F** | **6 F** |
| --- | --- | --- |
| **Sheath** | At the physician’s discretion.  Optional use of BIOTRONIK Fortress. | At the physician’s discretion.  Optional use of BIOTRONIK Fortress. |
| **Balloon (if applicable)** | Passeo.18 | At the physician’s discretion.  Optional use of BIOTRONIK Passeo series |
| **Stent (if applicable)** | Pulsar-18 | Self-expanding nitinol stent, at the physician’s discretion.  Optional use of BIOTRONIK Pulsar series |
| **Drug-coated balloon (if applicable)** | Ø ≤ 4.0mm Passeo-18  Ø > 4.0mm at the physician’s discretion | At the physician’s discretion.  Optional use of BIOTRONIK Passeo-18 Lux |
| **Closure device** | No vascular closure device should be used | At the physician’s discretion. |

**Supplementary Table 2: Baseline patient characteristics (secondary 4F cohort including the use of VCDs)**

|  | **4F-VCD**  **N=390** | **6F**  **N=404** | **p-value** |
| --- | --- | --- | --- |
| **Age, years** | 70 ± 11 | 69 ± 11 | 0.182 |
| **Male** | 282 (72.3) | 310 (76.7) | 0.152 |
| **Smoking** | 296 (75.9) | 310 (76.7) | 0.782 |
| **BMI** | N=378  26.8 ± 4.4 | N=396  27.0 ± 4.5 | 0.495 |
| **Hypertension** | 309 (79.2) | 326 (80.7) | 0.607 |
| **Hyperlipidaemia** | 233 (59.7) | 286 (70.5) | **0.001** |
| **Diabetes mellitus**  *Insulin dependent* | 116 (29.7)  *49 (12.6)* | 134 (33.2%)  *42 (10.4)* | 0.299 |
| **Renal insufficiency*** | 88 (22.6) | 65 (16.1) | **0.021** |
| **History of PAD** | 222 (56.9) | 243 (60.1%) | 0.356 |
| **Previous PVI/ surgeries** | 183 (46.9) | 196 (48.5%) | 0.654 |

Data are displayed as mean ± standard deviation or n (%). *according to site-assessment. BMI-body mass index, PAD-peripheral artery disease, PVI-peripheral vascular intervention, VCD-vascular closure device

**Supplementary Table 3: Baseline lesion characteristics (secondary 4F cohort including the use of VCDs)**

|  | **4F-VCD**  **N=561** | **6F**  **N=613** | **p-value** |
| --- | --- | --- | --- |
| **Lesion location**  Common femoral  SFA  Popliteal artery  BTK  Other | 25 (4.5)  318 (56.7)  81 (14.4)  107 (19.1)  30 (5.3) | 32 (5.2)  347 (56.6)  108 (17.6)  81 (13.2)  45 (7.3) | 0.543  0.979  0.139  **0.006**  0.163 |
| **Calcification***  Moderate  Heavy | N=555  112 (20.2)  114 (20.5) | N=607  179 (29.5)  107 (17.6) | **0.003** |
| **TASC classification**  A  B  C  D | N=556  138 (24.8)  189 (34.0)  136 (24.5)  93 (16.7) | N=607  154 (25.4)  234 (38.6)  129 (21.3)  90 (14.8) | 0.300 |
| **Thrombus present** | N=560  75 (13.4) | N=609  72 (11.8) | 0.419 |

Data are displayed as mean ± standard deviation or n (%). *according to site-assessment, BTK-below-the-knee, SFA-superficial femoral artery, VCD-vascular closure device

**Supplementary Table 4: Procedural characteristics (secondary 4F cohort including the use of VCDs)**

|  | **4F-VCD**  **N=390** | **6F**  **N=404** | **p-value** |
| --- | --- | --- | --- |
| **Femoral access** | N=399  399 (100.0) | N=410  407 (99.3) | 0.829 |
| **>1 vascular access** | 8 (2.1) | 5 (1.2) | 0.413 |
| **Access**  Antegrade  Retrograde | N=399  283 (70.9)  116 (29.1) | N=410  246 (60.0)  164 (40.0) | **0.001** |
| **Previous puncture at the same site** | N=399  48 (12.0) | N=410  36 (8.8) | 0.136 |
| **Devices used**  Plain balloon  Drug-coated balloon  Stent  Rotational thrombectomy  Atherectomy  Scoring balloon  Cutting balloon  Other | N=1053  542 (51.5)  112 (10.6)  384 (36.5)  0 (0.0)  0 (0.0)  0 (0.0)  4 (0.4)  11 (1.0) | N=1171  534 (45.6)  217 (18.5)  385 (32.9)  1 (0.1)  5 (0.4)  8 (0.7)  8 (0.7)  13 (1.1) | - |
| **Haemostasis**  VCD only  Compression device only  Manual compression only  VCD+compression device  VCD+manual compression  VCD+compression device+manual compression  Compression device+manual compression  Other combinations  None | 19 (4.9)  0 (0.0)  147 (37.7)  0 (0.0)  5 (1.3)  5 (1.3)  214 (54.9)  0 (0.0)  0 (0.0) | 202 (50.0)  0 (0.0)  24 (5.9)  23 (5.7)  91 (22.5)  36 (8.9)  25 (6.2)  1 (0.2)  1 (0.2) | **<0.0001** |
| **Haemostasis in ASC patients**, N=23  VCD only  Manual compression only  VCD+manual compression  VCD+compression device+manual compr.  Compression device+manual compression | N=12  1 (8.3)  6 (50.0)  1 (8.3)  0 (0.0)  4 (33.3) | N=13  1 (7.7)  0 (0.0)  3 (23.1)  8 (61.5)  1 (7.7) | **0.002** |
| **Manual compression time**, min | N=390  8.8 ± 9.1 [7.9;9.7] | N=402  4.2 ± 7.4 [3.5;4.9] | **<0.0001** |
| **Time to haemostasis**, min | N=390  13.4 ± 20.1 [11.4;15.4] | N=403  6.2 ± 8.9 [5.3;7.1] | **<0.0001** |
| **Procedure time**, min | N=388  39.8 ± 27.3 [37.1;42.5] | N=402  46.4 ± 27.6 [43.7;49.1] | **<0.0001** |
| **Procedure success** | 384 (98.5) | 400 (99.0) | 0.540 |

Data are displayed as mean ±standard deviation [95% confidence interval] or n (%). ASC-access-site complication, VCD-vascular closure device

**Supplementary Table 5: Clinical outcomes of patients for up to 30 days (secondary 4F cohort including the use of VCDs)**

|  | **4F-VCD**  **N=390** | **6F**  **N=404** | **p-value** |
| --- | --- | --- | --- |
| Discharge at day of procedure | 365 (93.6) | 382 (94.6) | **0.565** |
| Vascular closure device complication | 2 (0.5) | 10 (2.5) | **0.038** |
| **Access-site complications***  *Groin hematoma*  *Pseudoaneurysm*  *Groin-bleeding*  *AV-fistula*  *Arterial dissection*  *Thrombosis*  *VCD-related*  *Other* | N=385  12 (3.1)†  *5 (21.7)*  *5 (21.7)*  *1 (4.3)*  *0 (0.0)*  *0 (0.0)*  *1 (4.3)*  *1 (4.3)*  *10 (43.5)* | N=401  13 (3.2)  *4 (15.4)*  *6 23.1)*  *2 (7.7)*  *0 (0.0)*  *1 (3.8)*  *0 (0.0)*  *0 (0.0)*  *13 (50.0)* | 0.921  0.915 |
| **Major adverse events**  *Procedure- or device related death*  *Major target limb amputation*  *Clinically driven TLR* | N=384  7 (1.8)  *0 (0.0)*  *1 (0.3)*  *7 (1.8)* | N=402  8 (2.0)  *2 (0.5)*  *0 (0.0)*  *6 (1.5)* | **>0.999**  *0.500*  *0.490*  *0.785* |

Data are displayed as n (%). *could consist of several of the events below, †of these, 2 occurred in patients treated with VCDs. AV-ateriovenous, TLR-target lesion revascularization, VCD-vascular closure device

**Supplementary Table 6: Matched baseline and procedural characteristics**

|  | **4F**  **N = 310** | **6 F**  **N = 310** | **p-value** |
| --- | --- | --- | --- |
| **Age, years** | 69 ± 11 | 70 ± 11 | 0.732 |
| **Male** | 229 (73.9) | 233 (75.2) | 0.712 |
| **Smoking** | 237 (76.5) | 235 (75.8) | 0.851 |
| **BMI** | 26.9± 4.3 | 26.8 ± 4.4 | 0.903 |
| **Hypertension** | 250 (80.6) | 248 (80.0) | 0.840 |
| **Diabetes mellitus** | 90 (29.0) | 93 (30.0) | 0.792 |
| **Previous puncture at the same site** | 32 (10.3) | 32 (10.3) | >0.999 |
| **Critical limb ischemia** | 63 (20.3) | 61 (19.7) | 0.841 |
| **BTK** | 21 (6.8) | 16 (5.2) | 0.397 |
| **Moderate/ heavy calcifi-cation at puncture site** | 51 (16.5) | 53 (17.1) | 0.830 |
| **Access**  Antegrade  Retrograde | 207 (66.8)  103 (33.2) | 203 (65.5)  107 (34.5) | 0.734 |
| **Procedure time, min** | 40.4 ± 25.1 | 41.4 ± 23.5 | 0.458 |
| **Antiplatelet and anticoagulation therapy** | 293 (94.5) | 294 (94.8) | >0.999 |
| **High-enrolling site** | 114 (36.8 | 115 (37.1) | 0.934 |

Primary analysis population excluding patients with vascular closure device use in the 4F group. Data are displayed as mean ± standard deviation or n (%). BMI-body mass index, BTK-below-the-knee

**Supplementary Table 7: Adverse events on the day of procedure in patients with ambulatory failure**

| **4F-group** | | **6F-group** | | |
| --- | --- | --- | --- | --- |
| **Patient #** | **AEs on the day of the procedure** | **Patient #** | **AEs on the day of procedure** |  |
| 1 | Access-site complication | 1 | Access-site complication |  |
| 2 | Access-site complication | 2 | Access-site complication |  |
| 3 | Access-site complication | 3 | Access-site complication |  |
| 4 | Access-site complication, hypotension, bradycardia | 4 | Access-site complication |  |
| 5 | Small haematoma | 5 | Access-site complication |  |
| 6 | Small haematoma | 6 | Access-site complication |  |
| 7 | Rupture superficial femoral artery | 7 | Access-site complication |  |
| 8 | Exacerbation of chronic obstructive pulmonary disease, intermittent chest pain, bradycardia, hypokalaemia, minimal wound ooze after mobilisation | 8 | Access-site complication |  |
| 9 | Hypertension | 9 | Access-site complication, hypotension, bradycardia |  |
| 10 | Acute pulmonary oedema secondary to hypertension during the procedure | 10 | Slight oozing groin |  |
| 11 | Nausea | 11 | Slight oozing groin |  |
| 12 | Cardiac abnormalities | 12 | Oozing groin |  |
| - | - | 13 | Thrombus |  |
| - | **-** | 14 | Pain, no hematoma |  |
| - | - | 15 | Pain |  |

AE-adverse event

**Supplementary Table 8: Patients on anticoagulation and/or antiplatelet therapy**

|  | **4F**  **N=361** | **6 F**  **N=404** | **p-value** |
| --- | --- | --- | --- |
| **Baseline** | 339 (93.9%) | 382 (94.6%) | 0.701 |
| **Procedure** | 347 (96.1%) | 397 (98.3%) | 0.070 |
| **Discharge** | 356 (98.6%) | 404 (100.0%) | **0.023** |
| **30 days** | N=357  355 (99.4%) | N=401  396 (98.8%) | 0.457 |
